# Identification of Proteins in Promastigote and Amastigote-like *Leishmania* Using an Immunoproteomic Approach

**DOI:** 10.1371/journal.pntd.0001430

**Published:** 2012-01-17

**Authors:** Vinicio T. S. Coelho, Jamil S. Oliveira, Diogo G. Valadares, Miguel A. Chávez-Fumagalli, Mariana C. Duarte, Paula S. Lage, Manuel Soto, Marcelo M. Santoro, Carlos A. P. Tavares, Ana Paula Fernandes, Eduardo A. F. Coelho

**Affiliations:** 1 Departamento de Bioquímica e Imunologia, Instituto de Ciências Biológicas, Universidade Federal de Minas Gerais, Belo Horizonte, Minas Gerais, Brazil; 2 Programa de Pós-Graduação em Medicina Molecular, Universidade Federal de Minas Gerais, Belo Horizonte, Minas Gerais, Brazil; 3 Departamento de Patologia Clínica, Coltec, Universidade Federal de Minas Gerais, Belo Horizonte, Minas Gerais, Brazil; 4 Programa de Pós-Graduação em Ciências Saúde: Infectologia e Medicina Tropical, Faculdade de Medicina, Universidade Federal de Minas Gerais, Avenida Antônio Carlos, Belo Horizonte, Minas Gerais, Brazil; 5 Centro de Biología Molecular Severo Ochoa, CSIC, UAM, Departamento de Biología Molecular, Universidad Autónoma de Madrid, Madrid, Spain; 6 Departamento de Análises Clínicas e Toxicológicas, Faculdade de Farmácia, Universidade Federal de Minas Gerais, Belo Horizonte, Minas Gerais, Brazil; René Rachou Research Center, Brazil

## Abstract

**Background:**

The present study aims to identify antigens in protein extracts of promastigote and amastigote-like *Leishmania (Leishmania) chagasi* syn. *L. (L.) infantum* recognized by antibodies present in the sera of dogs with asymptomatic and symptomatic visceral leishmaniasis (VL).

**Methodology/Principal Findings:**

Proteins recognized by sera samples were separated by two-dimensional electrophoresis (2DE) and identified by mass spectrometry. A total of 550 spots were observed in the 2DE gels, and approximately 104 proteins were identified. Several stage-specific proteins could be identified by either or both classes of sera, including, as expected, previously known proteins identified as diagnosis, virulence factors, drug targets, or vaccine candidates. Three, seven, and five hypothetical proteins could be identified in promastigote antigenic extracts; while two, eleven, and three hypothetical proteins could be identified in amastigote-like antigenic extracts by asymptomatic and symptomatic sera, as well as a combination of both, respectively.

**Conclusions/Significance:**

The present study represents a significant contribution not only in identifying stage-specific *L. infantum* molecules, but also in revealing the expression of a large number of hypothetical proteins. Moreover, when combined, the identified proteins constitute a significant source of information for the improvement of diagnostic tools and/or vaccine development to VL.

## Author Summary

Canine visceral leishmaniasis (CVL) is an important emerging zoonosis caused by *Leishmania (Leishmania) infantum* in the Mediterranean and Middle East and *L. (L.) chagasi* (syn. *L. (L.) infantum*) in Latin America. Due to their genotypic relationships, these species are considered identical. The present study focused on comparing the protein expression profiles of the promastigote and amastigote-like stages of *L. infantum*, by means of a protein separation by two-dimensional electrophoresis and identification by mass spectrometry. The present study attempted to identify proteins recognized by antibodies present in the sera of dogs with asymptomatic and symptomatic visceral leishmaniasis. A total of one hundred and four proteins were identified. Of these, several stage-specific proteins had been previously identified as diagnosis and/or vaccine candidates. In addition, antibodies from infected dogs recognized thirty-one proteins, which had been previously considered hypothetical, indicating that these proteins are expressed during active infection. Therefore, the present study reveals new potential candidates for the improvement of diagnosis of CVL.

## Introduction

Visceral leishmaniasis (VL) is an important parasitic disease, with a worldwide distribution in 88 countries, where a total of 350 million people may be at risk. In Brazil, the disease is an endemic zoonosis caused by the parasitic protozoa *Leishmania (Leishmania) chagasi* syn. *L. (L.) infantum* [1]. Dogs are the main parasite domestic reservoirs, and culling of seropositive dogs, as detected by means of serological tests using promastigote antigens, *i.e.* RIFI or ELISA, is a major VL control measure adopted in Brazil. Therefore, to reduce the transmission of parasites between dogs and humans, it is necessary, among other aspects, to diagnose canine visceral leishmaniasis (CVL) as early as possible, by means of sensitive and specific diagnostic tools [2], [3].

Upon infection, dogs develop three different stages of the disease: symptomatic, oligosymptomatic, and asymptomatic [4]. Symptomatic infections tend to evolve into animal deaths, and their clinical manifestations include cutaneous alterations, such as alopecia, dermatitis, and onychogryphosis [5], [6], as well as visceral dysfunctions in the kidneys, liver, and heart [7], [8]. A high number of infected dogs remain asymptomatic and present low levels of specific antibodies; however, some dogs do in fact develop a few mild symptoms, which are classified as oligosymptomatic [4].

Routine diagnosis of leishmaniasis has been based on classic parasitological methods, where infected skin tissue and aspirates, or biopsy specimens of visceral tissues (*i.e.*, spleen, liver, and bone marrow), undergo microscopic examinations and cultures [9]. Classic serological methods are limited by low sensitivity and/or specificity of the tests, requiring repeated tissue sampling and a trained laboratory staff [10]. The diagnosis of CVL, by means of ELISA, based on *Leishmania* antigens has shown variable values of sensitivity and/or specificity, mainly due to antigenic similarities between *Leishmania* and other protozoa [10]. As a strategy to develop a more specific test, several parasite antigens have been tested in prior studies [11]–[14]; however, due to frequent low specificity and sensitivity in detecting asymptomatic infections and the high variability observed in the humoral response of individual infected dogs [15], it has been postulated that an efficient diagnosis may require a mixture of antigens or the use of chimerical antigens [16]–[19].

Proteomic approaches applied to study *Leishmania* protein expression patterns offer the possibility to assign potential functions for proteins, including those previously identified by genomics as hypothetical, new diagnostic markers, vaccine candidates, and/or potential drug targets [20]–[23]. Several proteomic studies have been performed to study stage-specific expression and differentiation in *Leishmania* [24]–[32]. The coupling of antibodies specific to parasite antigens generated during different stages of disease progression in dogs will certainly contribute to refining this analysis, which aims to identify not only differentially expressed proteins, but also potentially new antigens identified by the immune system during active infection. Recently, the discovery of antigens through proteomics has been indicated as one of the main research priorities for further development and improvement of leishmaniasis vaccines [33].

In this work, an immunoproteomic approach, together with two-dimensional electrophoresis (2DE) and mass spectrometry, was carried out to analyze the protein expression profiles of promastigote and amastigote-like *L. infantum*. Aimed at identifying new diagnostic markers and/or vaccine candidates, antibodies present in the sera of dogs with asymptomatic and symptomatic VL were added to this analysis, allowing for the identification of several known, as well as hypothetical, *L. infantum* antigenic proteins.

## Materials and Methods

### Parasite culture

Experiments were carried out using the *Leishmania (Leishmania) chagasi* syn. *L. (L.) infantum* (MHOM/BR/1970/BH46) strain. The stationary phase of promastigote cells were grown at 24°C in Schneider's medium (Sigma, St. Louis, MO, USA), supplemented with 20% inactivated fetal bovine serum (FBS, Sigma), 20 mM L-glutamine, 200 U/mL penicillin, and 100 µg/mL streptomycin, at pH 7.2, as previously described [34]. The amastigote-like cells were obtained as described by Doyle et al. (1991) [35].

### Sera samples

The present study used sera samples from 60 *L. infantum*-infected dogs (40 clinically symptomatic and 20 asymptomatic) from Belo Horizonte, Minas Gerais, Brazil. Animals were considered symptomatic when three or more of the following symptoms were present: loss of weight, hepatomegaly, alopecia, adenopathy, onychogryposis, conjunctivitis, and exfoliative dermatitis on the nose, tail, or ear tips. The asymptomatic animals were free from clinical symptoms. All sera samples from either symptomatic or asymptomatic animals were positive when tested by RIFI and ELISA, and the presence of amastigote stage of the parasite was confirmed by microscopic observation and *in vitro* culture using aspirates from popliteal and/or prescapular lymphoid nodes or bone marrow and/or tissue fragments. The control group consisted of sera from 20 dogs living in non-endemic areas from VL, with no clinical signs or suspicion of leishmaniasis, and which showed negative parasitological and serological tests. Sera samples used in this study were kindly provided by Dr. Maria Norma Melo (Departmento de Parasitologia, Instituto de Ciências Biológicas, UFMG).

### Preparation of protein extracts

The protein extraction from promastigote and amastigote-like stages *L. infantum* and 2DE were performed following a modified protocol [36]. Briefly, cells from both stages (1×10^10^ cells) were washed three times in 40 mM Tris-HCl, pH 7.2, by centrifugation at 5000× *g* for 10 min at 4°C. The pellets were resuspended in lyses buffer solution [7 M urea, 2 M thiourea, 4% chol-amidopropyl dimethylammonio-1-propanesulfonate (CHAPS), 40 mM dithiothreitol (DTT), 2% IPG buffer (pH 4–7), 40 mM Tris], and a protease inhibitor cocktail (GE Healthcare, Upsala, Sweden) was added. Samples were incubated for 1 h at room temperature, with occasional vortexing. Purification was carried out by protein precipitation using a 2D Clean UpKit (GE Healthcare), according to manufacturer instructions. Whole cell extracts were measured by a 2D Quant-Kit (GE Healthcare), and aliquots were immediately frozen at −80°C, until use.

### Isoeletric focusing (IEF)

For the first-dimension electrophoresis, 150 µg of protein extract was added to a volume of 250 µL with a rehydration solution [7 M urea, 2 M thiourea, 2% CHAPS, 40 mM DTT, 2% immobilized pH gradient (IPG-buffer, pH 4–7, trace bromophenol blue)]. Next, samples were applied to IPG strips (13 cm, pH 4–7; GE Healthcare) for passive rehydration overnight at room temperature. After in-gel rehydration for 12 h, isoeletric focusing was performed at 500 V for 1 h, 1.000 V for 1 h, and 8.000 V for 8 h, using a Multiphor II electrophoresis unit and EPS 3500 XL power supply (Amersham, Piscataway, NJ, USA).

### SDS-PAGE

After IEF, each strip was incubated for 15 min in a solution made up of 10 mL of a 50 mM Tris-HCl buffer pH 8.8, 6 M urea, 30% (v/v) glycerol, 2% (w/v) SDS, 0.002% bromophenol-blue, and 125 mM DTT, followed by a second incubation step in the same buffer solution, excluding DTT, which was replaced by 125 mM iodacetamide. IPG strips were transferred to a 12% polyacrilamide and sealed with agarose solution (agarose and bromophenol blue in a Tris-glicine cathode buffer). The protein standard was purchased from Invitrogen (BenchMark Protein Ladder). Electrophoresis was performed in a Mini-Protean II system (BioRad) connected to a MultiTemp II cooling bath (Amersham Biosciences), in a Tris/glycine/SDS buffer. Proteins were separated at 200 V, until the dye front had reached the bottom of the gel.

### Immunoblotting 2DE analysis and protein identification

To identify the reactive spots that were recognized by the antibodies present in the sera samples from asymptomatic and/or symptomatic CVL, Western blot analyses were performed. Whole cell extracts of promastigote and amastigote-like *L. infantum* were separated electrophoretically and transferred onto cellulose membranes (Schleicher & Schull, Dassel, Germany) by semi-dry blotting for 2 h at 400 mA. Membranes were blocked in 5% (w/v) low-fat dried milk in TBS 1× (pH 7.4) plus 0.05% Tween 20 for 2 h at room temperature. Next, the membranes were washed 6 times (10 min each) with the blocking solution and pre-incubated in a pool of sera of symptomatic or asymptomatic CVL (1∶200 diluted) for 2 h at room temperature. Then, membranes were incubated with a peroxidase-conjugated goat anti-dog IgG secondary antibody (1∶5.000 diluted) for 2 h at room temperature. After having been washed 3 times with TBS 1× plus 0.5% Tween 20, immunoblots were developed, using a solution made up of chloronaphtol, diaminobenzidine and H_2_O_2_. To select and identify the spots recognized by antibodies of CVL sera, three independent protein preparations, each obtained from independent parasite cultures, were performed. The 2DE gels were stained with colloidal Coomassie Brilliant Blue G-250, following procedures described by Neuhoff et al. (1988) [37]. For image analysis, the stained gels were scanned using an ImageScanner III (GE Healthcare). Reactive spots recognized by antibodies in the sera samples of asymptomatic and/or symptomatic CVL were excised manually from the gels for protein identification.

### Protein digestion, peptide extraction, and spot handling

Spots were manually excised, and fragments were washed in 25 mM ammonium bicarbonate/50% acetonitrile until completely destained. After drying, gel fragments were placed on ice in a 50 µL protease solution (20 ng/mL of a sequence grade-modified trypsin in a 25 mM ammonium bicarbonate) (Promega Biosciences, CA, USA), for 30 min. Excess protease solution was removed and replaced by 25 mM ammonium bicarbonate. Digestion was performed at 37°C for 18 h. Peptide extraction was performed twice for 15 min, using 30 µL of 50% acetonitrile/5% formic acid. Trypsin (Promega) digests were concentrated in a Speed-Vac (Savant, USA) to approximately 10 µL and desalted using Zip-Tip (C18 resin; P10, Millipore Corporation, Bedford, MA, USA). Samples were mixed with a matrix (5 mg/ml recrystallized α-cyano-4-hydroxycinnamic acid) in a volume of 1 mL (1∶1 ratio) and then spotted for MALDI-TOF/TOF Ultraflex III (Bruker, Daltonics, Germany).

### Protein identification and database search

To determine the MS spectrum of the immunoreactive spots, the digests were spotted onto 600 µm Anchorchips (Bruker Daltonics). Spotting was achieved by pipetting, in duplicate, 1 µL of analyte onto the MALDI target plate, then adding 5 mg/mL α-cyano-4-hydroxycinnamic acid diluted in 3% TFA/50% acetonitrile, which contained 2 mM ammonium phosphate. The Bruker peptide calibration mixture was spotted down for external calibration. All samples were allowed to air dry at room temperature, and 0.1% TFA was used for on-target washing. All samples were analyzed in the positive-ion, reflection mode, through a MALDI-TOF/TOF Ultraflex III mass spectrometer (Bruker, Daltonics, Germany). Each spectrum was produced by accumulating data from 200 consecutive laser shots, with a frequency of 100 Hz, and an m/z range of 1.000–4.000. Instrument calibration was achieved by using peptide calibration standard II (Bruker Daltonics), a mixture of angiotensin I & II, substance P, bombesin, ACTH clip 1–17, ACTH clip 18–39 and somatostatin 28, as the internal standard. Peptide masses were measured as mono-isotopic masses. The MS peaks with the highest intensities were selected for MS/MS fragmentation analyses.

The resulting spectra were processed using Flex analysis software, version 2.4 (Bruker Daltonics), with the following settings: peak detection algorithm set at SNAP (Sort Neaten Assign and Place), S/N threshold at 3, precursor and product ion tolerances were set at 0.5 Da, and quality factor threshold at 50. The trypsin autodigestion ion peaks (842.51, 1045.56, 2211.10, and 2225.12 Da) were used as internal standards to validate the external calibration procedure. Matrix, and/or autoproteolytic trypsin fragments, and known contaminants (*i.e.*, keratins) were manually removed. The resulting peptides list was used to search in the NCBI database (http://blast.ncbi.nlm.nih.gov) for the organism option of *Leishmania* (taxid:5658). According to the obtained results, and using the peptide sequences identified for each protein, the following parameters were used as selection criteria: total score, query coverage, and *E* value. Poor quality spectra were not considered for selection in the protein sequence database.

## Results

### 2DE protein maps of promastigote and amastigote-like total extracts of *Leishmania infantum*

**Figure 1:**
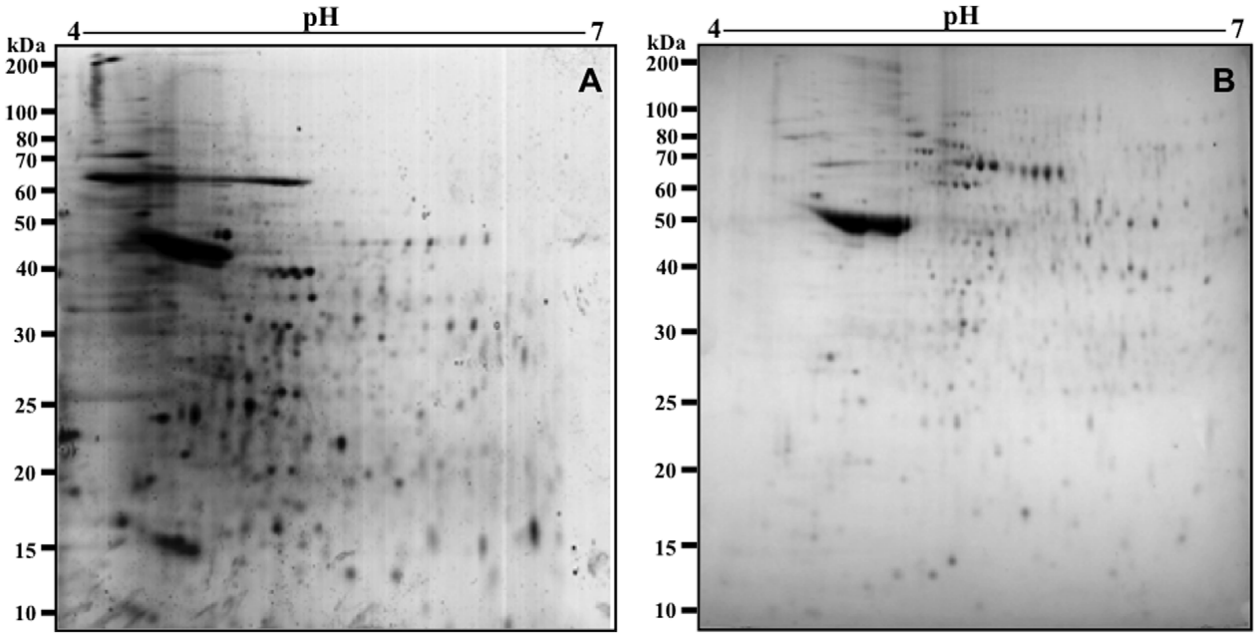
Two-dimensional profiles of the total extracts from *Leishmania infantum* promastigote and amastigote-like stages. 2DE gels were obtained after separation of promastigote (in A) and amastigote-like (in B) protein extracts (150 µg, each one) by 2DE (first dimension: IEF pH range 4–7, second dimension: 12% SDS-PAGE), and staining with colloidal Coomassie Brilliant Blue G-250. 2DE gels were derived from three independent protein preparations. One representative preparation of each parasite stage was used in this study.

Electrofocusing on pH 4–7 IPG strips, approximately 350 protein spots in *L. infantum* promastigote and 200 spots in amastigote-like stages could be observed clearly (Figure 1). Promastigote stages, as compared to amastigote-like forms, presented a larger number of visible spots, and differences could be observed in the molecular weights of the band profiles obtained between them: most of the promastigote spots were found between 15 and 50 kDa (Figure 1A), while in the amastigote-like stage, these bands tended to be distributed between 25 and 70 kDa (Figure 1B). The 2DE spot profiles obtained from promastigote and amastigote-like were highly reproducible in terms of both the total number of protein spots and their relative positions and intensities in the three 2DE gels performed in this study (data not shown).

### Immunoblotting analysis of 2DE maps of promastigote and amastigote-like stages of *Leishmania infantum*

**Figure 2:**
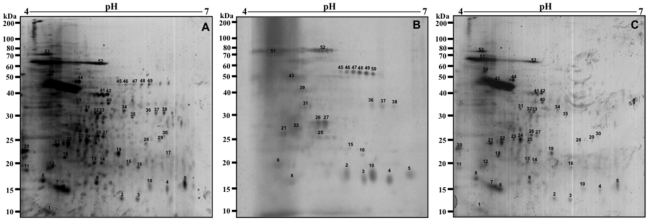
Immunoproteomic analyses of the protein extract from the *Leishmania infantum* promastigote stage. 2DE gels obtained after separation of total protein extract (150 µg) of promastigote stage by 2DE (first dimension: IEF pH range 4–7, second dimension: 12% SDS-PAGE), and staining with colloidal Coomassie Brilliant Blue G-250 (A, as described in Figure 1). Immunoblots of reactive spots were identified after incubation of the membrane with pools of sera of asymptomatic (B) or symptomatic (C) VL dogs. Bound antibodies were detected with goat anti-dog IgG antibodies at a 1∶5.000 dilution. The x-axis represents the tentative isoeletric point (*pI*), while the y-axis represents the approximate molecular weight (kDa) as determined by a commercial 2DE gel marker (BenchMark Protein Ladder). Protein spots were numbered, and their identities are given in Figure 5. Immunoblots are a reliable representation of three independent experiments.

To investigate the antigenicity of proteins in the *L. infantum* promastigote stage, immunoblots were performed, using a pool of symptomatic and asymptomatic VL dogs' sera. Using the profile obtained from the 2DE gel as a comparison (Figure 2A), the pool of sera from asymptomatic VL dogs reacted by presenting approximately 40 protein spots in the promastigote extract (Figure 2B), whereas when the pool of sera from symptomatic VL dogs were used, the immunoblots revealed approximately 80 protein spots (Figure 2C).

**Figure 3:**
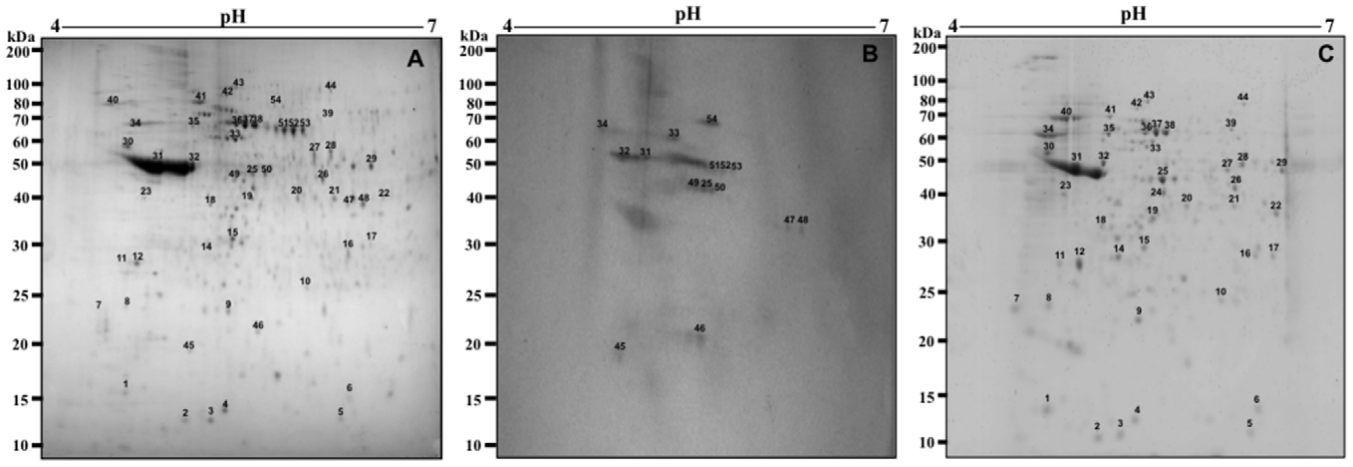
Immunoproteomic analyses of the protein extract from the *Leishmania infantum* amastigote-like stage. 2DE gels obtained after the separation of total protein extracts (150 µg) of amastigote-like stages by 2DE (first dimension: IEF pH range 4–7, second dimension: 12% SDS-PAGE), and staining with colloidal Coomassie Brilliant Blue G-250 (A, as described in Figure 1). Immunoblots of reactive spots were identified after incubation of the membrane with pools of sera of asymptomatic (B) or symptomatic (C) VL dogs. Bound antibodies were detected with goat anti-dog IgG antibodies at a 1∶5.000 dilution. The x-axis represents the tentative isoeletric point (*pI*), while the y-axis represents the approximate molecular weight (kDa) as determined by a commercial 2DE gel marker (BenchMark Protein Ladder). Protein spots were numbered, and their identities are listed in Figure 6. Immunoblots are a reliable representation of three independent experiments.

In this same manner, using the 2DE gel profile obtained of amastigote-like extract for comparison (Figure 3A), the sera of asymptomatic VL dogs reacted by presenting approximately 30 protein spots (Figure 3B), whereas when the sera from VL symptomatic dogs were used, immunoblots revealed approximately 70 protein spots (Figure 3C). It is important to note that how a pool of sera of symptomatic (n = 40) or asymptomatic (n = 20) VL dogs was used in the experiments, the individual variability in the humoral responses did not bias the reactivity observed in the immunoblotting analysis. As a control, the different 2DE gels and immunoblots applied to promastigote and amastigote-like extracts were probed with sera of control dogs presenting negative parasitological, clinical, and serological analyses, and no protein spot could be detected in either case (data not shown).

**Figure 4:**
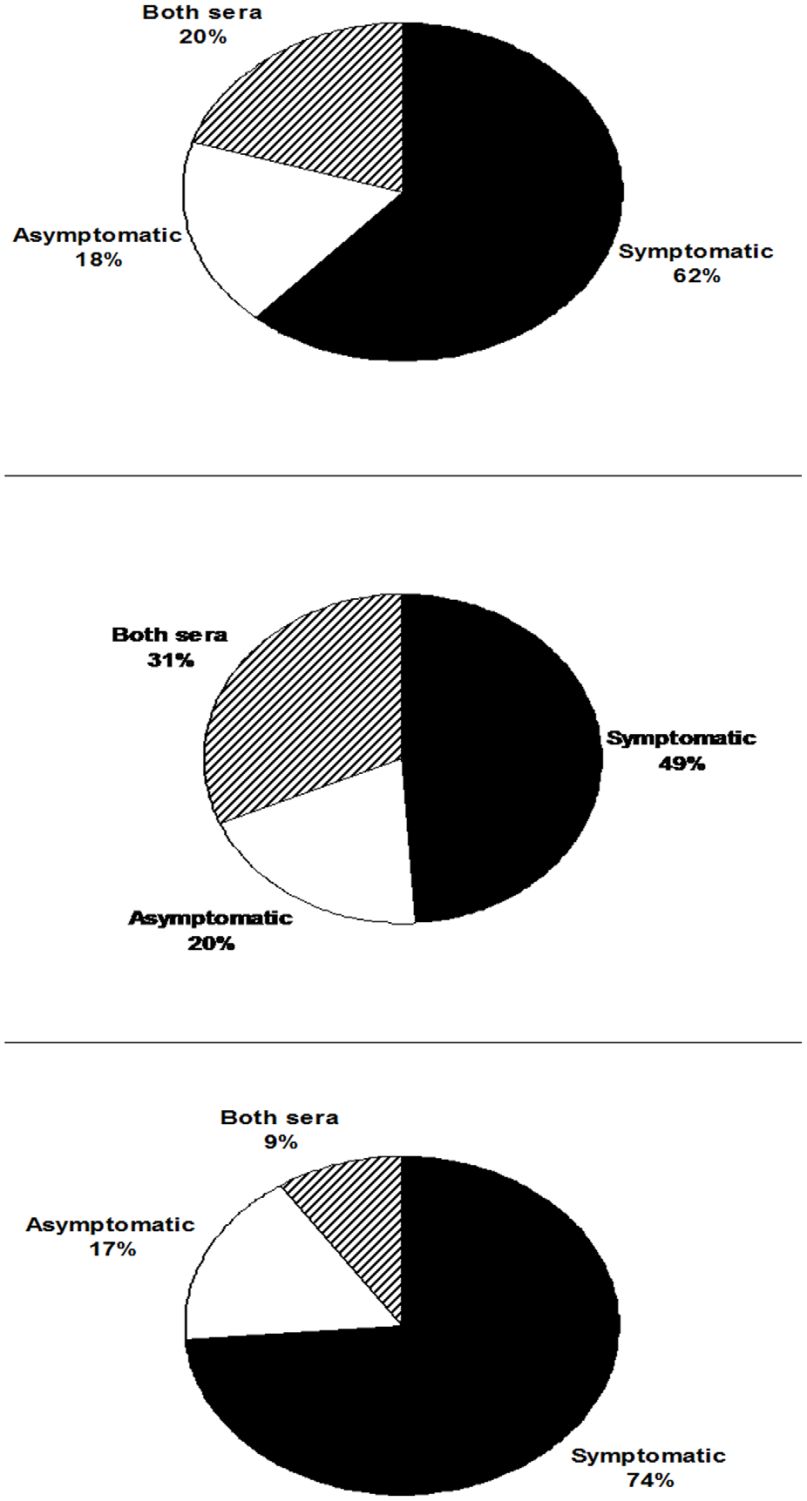
Comparison of spots identified in protein extracts from promastigote and amastigote-like stages of *Leishmania infantum*. Diagrams show the percentage of protein spots identified in either individual or combined parasite stages. In A, the percentage of total proteins identified by asymptomatic (19/18%), symptomatic (64/62%), and a combination of both sera classes (21/20%). In B, the percentage of proteins from the promastigote stage identified by asymptomatic (10/20%), symptomatic (25/49%), and a combination of both sera classes (16/31%). In C, the percentage of proteins from amastigote-like stage identified by asymptomatic (9/17%), symptomatic (39/74%), and a combination of both sera classes (5/9%).

In Figure 4, the diagram shows that, from a total of 104 (100%) proteins in both promastigote and amastigote-like extracts, 64 (62%) could be identified by the sera of symptomatic CVL, while the sera of asymptomatic animals detected that 19 (18%) and 21 (20%) proteins proved to be reactive in both classes of sera, respectively. Of the proteins identified in promastigote antigenic extracts, the sera of symptomatic and asymptomatic VL dogs, as well as the combination of both sera, could identify approximately 49%, 20%, and 31% of the proteins, respectively. In amastigote-like extract, the sera of asymptomatic and symptomatic VL animals and the combination of both sera classes identified approximately 74%, 17%, and 9% of the proteins, respectively.

### Identification of *Leishmania infantum* promastigote proteins by MS/MS and the use of protein databases

**Figure 5:**
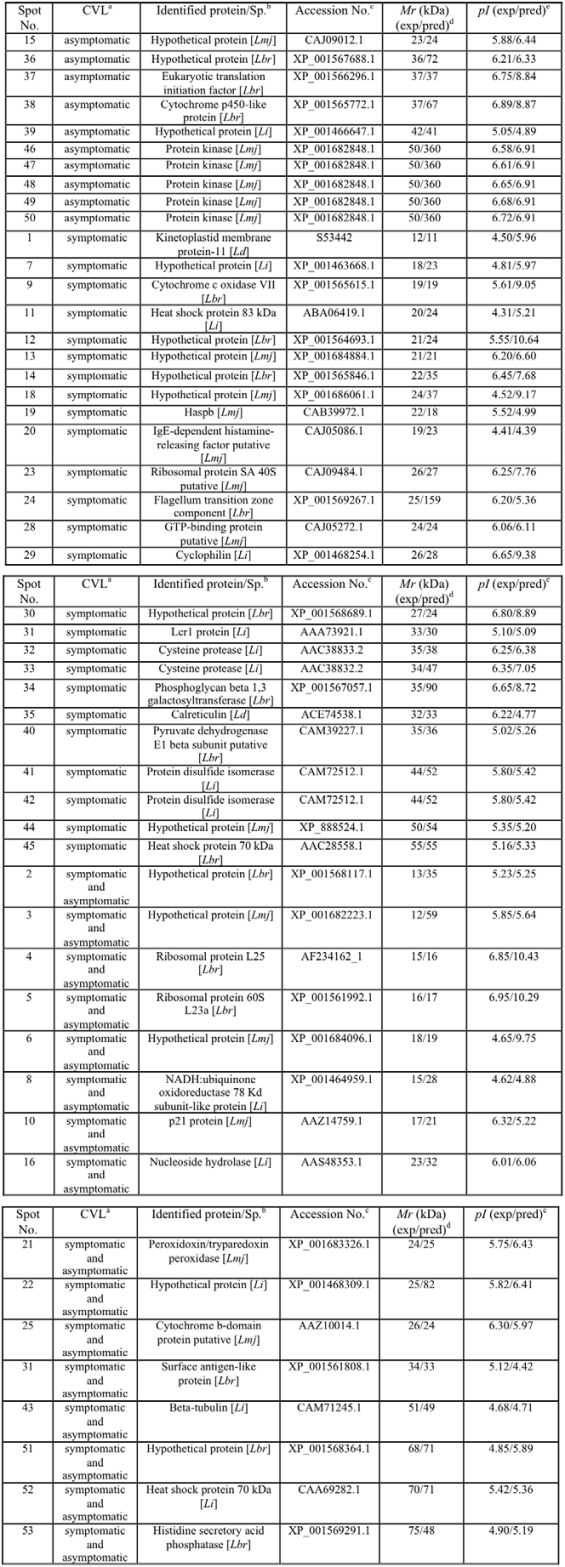
Proteins of *Leishmania infantum* promastigotes identified by an immunoproteomic approach. a) Sera samples of dogs with VL. b) Name of the identified protein and species: Lmj, *L. major*; Lbr, *L. braziliensis*; Li, *L. infantum*; Ld, *L. donovani*. c) Accession numbers according to NCBI. d) Experimental expected and predicted molecular weights (*M*r, in KDa). e) Experimental expected and predicted isoeletric points (p*I*).

In an attempt to establish a reference map of identified spots using the sera of asymptomatic and symptomatic VL dogs in immunoblots with *L. infantum* promastigote stage, 51 well-identified spots were used (25, 10, and 16 identified by symptomatic, asymptomatic, and the combination of both sera classes, respectively). Reactive spots were selected and excised from 2DE gels for analyses by mass spectrometry, as described in the Materials and Methods Section. Results are summarized in Figure 5.

Among the proteins recognized by the sera of symptomatic VL dogs, 7 hypothetical and 18 known proteins, which included cysteine proteinases, heat shock proteins (HSP70 and HSP83), and other proteins related to parasite virulence, such as disulfide isomerase [38], [39], cyclophilin [40], and cytochrome *c* oxidase VII [41], [42] were detected. Possible targets for therapeutic interventions, such as GTP-binding protein; proteins already characterized for diagnosis, such as KMP-11 [43], [44] and calreticulin [45]; and vaccine candidates, such as KMP-11 [46] and Lcr1 protein [47], were also observed. Using the sera of asymptomatic VL dogs, 3 hypothetical and another 7 known proteins were detected, including a protein kinase, elongation factor (eIFE), and cythcrome p450, which have been considered therapeutic targets for leishmaniasis [48]–[51]. Five hypothetical proteins could be identified by both sera classes, whereas among the proteins with identifiable functions, some have been previously evaluated as candidates for the diagnosis and/or vaccine for leishmaniasis, such as nucleoside hydrolase [52], ribosomal proteins [53], [54], peroxidoxin [55], and β-tubulins [56], [57].

### Identification of *Leishmania infantum* amastigote-like proteins by MS/MS and the use of protein databases

**Figure 6:**
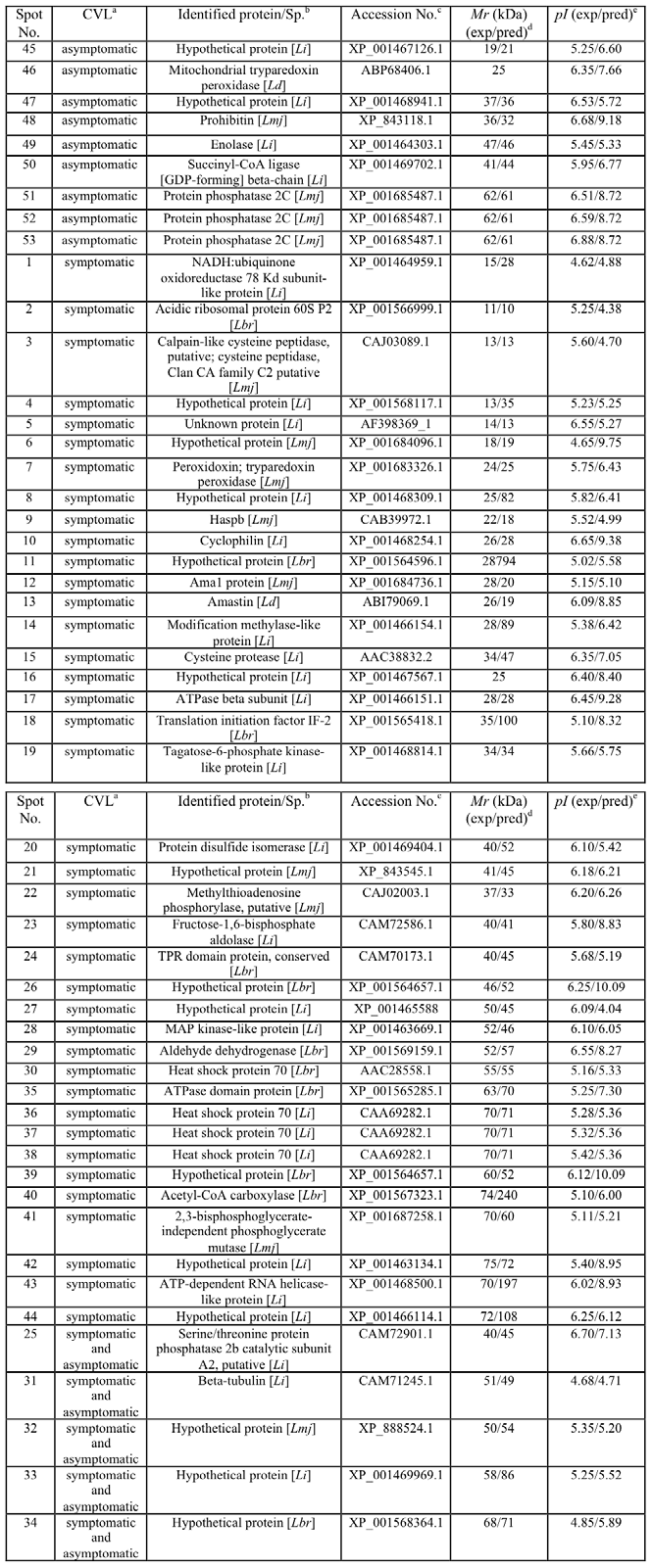
Proteins of *Leishmania infantum* amastigotes-like identified by an immunoproteomic approach. a) Sera samples of dogs with VL. b) Name of the identified protein and species: Lmj, *L. major*; Lbr, *L. braziliensis*; Li, *L. infantum*; Ld, *L. donovani*. c) Accession numbers according to NCBI. d) Experimental expected and predicted molecular weights (*M*r, in KDa). e) Experimental expected and predicted isoeletric points (p*I*).

Due to the importance of the amastigote life cycle in leishmaniasis, this parasite stage cultured in axenic conditions was immunoblotted with the sera of asymptomatic and symptomatic VL dogs. The analysis of approximately 200 protein spots allowed for the identification of 53 well-defined proteins that were recognized by the sera of asymptomatic and symptomatic CVL; with 39, 9, and 5 identified by symptomatic, asymptomatic, and the combination of both sera classes, respectively. Similar to the study with promastigote stage, reactive spots were selected and excised from 2DE gels for identification. It is worth noting that A2 [13], ATP-dependent RNA helicase [58], and amastin [59] proteins were identified only in the amastigote-like extract, since these proteins are characterized as amastigote-specific in *Leishmania* (Figure 6).

When the sera of asymptomatic VL dogs were used against amastigote-like stage, 2 hypothetical and 7 known proteins, including the phosphatase 2C protein [60], virulence factors (prohibitin) [61], diagnosis markers, vaccine candidates (mitochondrial tryparedoxin peroxidase) [62], and drug targets (succinyl-coA ligase [GDP-forming] β-chain) [63] could also be identified.

A significant number of proteins (39 of 53) were found to be present in both stages and to react with sera of symptomatic VL dogs. Several of these are linked to housekeeping metabolism pathways, such as protein synthesis or cellular stress, and included ribosomal proteins, cyclophilin, Haspb, cysteine proteinases, eIFE, and heat shock proteins [30], [53], [54]. In addition, some proteins involved in parasite virulence, such as fructose-1,6-biphosphatealdolase (aldolase) [64], [65], as well as therapeutic targets, such as ATPase β-subunit [66], cysteine peptidases [67], and methylthioadenosine phosphorylase [68], could also be identified.

## Discussion

The present work applied an immunoproteomic approach in *L. infantum* promastigote and amastigote-like antigenic extracts, using pools of sera of asymptomatic and/or symptomatic VL dogs, in an attempt to compare their protein expression profiles and identify new targets for future immunological applications of VL. The use of pools of sera of both asymptomatic and symptomatic VL dogs in this study appears to have reduced the impact of individual animal immune response variations on *L. infantum* antigens.

The life cycle and the clonal propagation of *Leishmania* represent particular problems to obtain homogeneous populations of parasites to use in comparative proteomic analyses. In addition, it is difficult to purify amastigote-like stages from host tissues and, in general, contamination with host proteins is an important drawback to be overcome. Although axenic amastigotes display many of the features of *in vivo* intracellular parasites, a constant concern among researchers has been the extent to which axenic amastigotes resemble the intracellular parasites [69], [70].

The present study employed the protocol described by Doyle et al. (1991) [35] to obtain amastigote-like stages of *L. infantum*. Carvalho et al. (2002), using the same protocol in amastigote-like stage, demonstrated the expression of the amastigote-specific A2 protein in *L. chagasi* and *L. amazonensis* [13], by applying Western blot experiments using an A2-specific monoclonal antibody. In the present work, A2 and two other amastigote-specific proteins – ATP-dependent RNA helicase [58] and amastin [59] – were detected in the immunoblots. The expression of these proteins by the axenic amastigotes suggests that they are, at least in part, comparable to tissue amastigotes and their gene expression, which is in accordance with the approach used in the present study to identify amastigote-specific antigens. Conversely, some proteins that are known to be specific, or that are more highly expressed in promastigotes, such as the flagellum transition zone component and the phosphoglycan beta-1,3-galactosyltransferase, which is linked to LPG synthesis, could only be detected in immunoblots of promastigote antigenic extracts.

As expected, some of the proteins identified in the present work have been previously associated with humoral responses in VL and are candidate antigens for diagnosis. Curiously, Haspb, a protein identified in promastigote extracts, presents a high homology, together with a family of related hydrophilic, kinesin antigens of *Leishmania spp.*, which includes the K26 and K39. These antigens were largely tested and used in serological diagnosis of VL, although they have been reported to be more sensitive for the diagnosis of symptomatic dogs [10], [16], [71].

The evolution from an asymptomatic to a symptomatic disease is largely dependent on host immune responses. Immunopathogenesis of CVL has been associated with two major responses: a Th1 immune response is linked to the control of infection and a predominant, although not polarized, Th2 response responsible for the development of a patent disease [72]. Here, several proteins proved to be reactive when in contact with sera of asymptomatic animals, a stage of infection in which dogs developing immune responses able to control parasite replication. Although humoral responses cannot be correlated directly with protection, IgG1 and IgG2 responses are largely T-cell dependent. Moreover, IgG2 antibodies have been commonly associated with protective immune responses and IFN-γ production [73]. Therefore, parasite antigens that react with antibodies from asymptomatic animals, in addition to their potential as diagnostic antigens, may be associated with protective responses and may well represent potential vaccine candidates.

In addition, the use of pools of sera of both asymptomatic and symptomatic VL dogs in the present study implies that no immune response variations by individual animals to *L. infantum* antigens could be observed. Due to the high degree of variability found in the humoral responses to different parasite antigens in CVL sera [16], the results give rise to the possibility of obtaining new recombinant antigens and analyzing their properties as tools for the diagnosis of all forms of CVL.

Predominant proteins in the *pI* 4–7 2DE gels presented a molecular mass range of between 15 and 50 kDa for promastigote stage and of 25 to 70 kDa for the amastigote-like stage. These results are in agreement with findings from Dea-Ayuela et al. (2006) [74], who identified approximately 700 spots in promastigote extracts, with molecular masses similar to those found in the present study. By contrast, Brotherton et al. (2010) reported, for the first time, several highly basic proteins in both amastigote and promastigote protein extracts, which were enriched by coupling fractionation by *pI* with free-flow electrophoresis in their proteomic analysis of stage-specific expressions of *L. infantum* [32]. Therefore, the selection of a *pI* 4–7 range may have limited our analysis.

In addition, the presence of elongation factors; heat shock proteins, such as HSP70, HSP83, and other chaperones; as well as tubulin and other housekeeping proteins, among the most abundantly detected in both antigenic extracts, were in good agreement with other studies and present a reliable validation of the immunoproteomic analysis performed herein [56], [57]. Some proteins detected in *Leishmania* extracts could be found in multiple spots or as proteolytic fragments. In addition, protein degradation cannot be completely discarded, although the protein extracts were obtained in the presence of a cocktail of protease inhibitors. However, this finding may also be associated with the presence of isoforms or to the extensive post-translational modification and processing of proteins, known to occur in *Leishmania sp*., and as previously observed in other proteomic analyses [32].

The analysis of the three available *Leishmania* species genomes (*L. braziliensis*, *L. major*, and *L. infantum*) revealed that they are highly conserved at both nucleotide (less than 1% species-specific genes) and aminoacid levels (77 to 92%), although it has been estimated that *Leishmania* species have evolved from a common ancestor as far as 15–50 million years ago [75]. Although *Leishmania* has a digenetic life cycle with significant biochemical and morphological alterations, it has been estimated that only 0.2% to 13.0% of these genes show preferential stage-specific expression [76]. Therefore, there is no consensus on the number of genes that are differentially expressed in different stages, and discrepancies are likely due to the design of the genomic and cDNA arrays used in different studies [77]. Nevertheless, protein expression levels showed a weak correlation with gene expression levels [29], [75], which could be linked to post-transcriptional events. In this context, proteomic studies are crucial and may reveal how *Leishmania* uses this conserved genetic background to generate protein variability, an alternation of stages during its life cycle, and to cause rather distinct diseases.

Tests based on serological techniques to diagnose human and canine VL are facilitated by the strong humoral response that accompanies the infection by viscerotropic *Leishmania* species [78]. Nonetheless, detection of asymptomatic dogs may be critical to control epidemics and to avoid the spread of the disease among dogs, as well as between dog and human populations [4], [5], [79]. However, total and soluble *Leishmania* antigen-based ELISA fails to detect a great percentage of asymptomatic cases of the disease [13], [80]. Similar findings have also been reported for recombinant antigens [16]. Therefore, there is still space to identify new antigens capable, whether alone or in combination, of improving the serological diagnosis of CVL. In this sense, the present study represents a step forward in the proteomic analysis of *Leishmania* species since, in addition to known antigenic stage-specific proteins, a high number of hypothetical proteins of *L. infantum* were also identified. Altogether, these proteins warrant further investigation in an attempt to potentially improve diagnosis. The fact that antibodies present in the pools of sera of infected dogs identified hypothetical proteins indicates that these proteins are expressed during active infection. Therefore, the data obtained in the present study represent not only a contribution toward the future improvement of diagnostic tools and vaccines for CVL, but also a step towards a better understanding of the biological role of these proteins in *L. infantum* metabolism, virulence, and pathogenesis. Thus, additional studies are most certainly encouraged.
